# Expression of CXCL12 receptors in B cells from Mexican Mestizos patients with systemic lupus erythematosus

**DOI:** 10.1186/1479-5876-10-251

**Published:** 2012-12-18

**Authors:** Vincent Biajoux, Alexandre Bignon, Christelle Freitas, Valérie Martinez, Marcus Thelen, Guadalupe Lima, Juan Jakez-Ocampo, Dominique Emilie, Luis Llorente, Karl Balabanian

**Affiliations:** 1 Université Paris-Sud, Laboratoire "Cytokines, Chimiokines et Immunopathologie", UMR_S996, Clamart, France; 2 INSERM, Laboratory of Excellence in Research on Medication and Innovative Therapeutics (LERMIT), Clamart, France; 3 Service de Médecine Interne et d’Immunologie Clinique, AP-HP, Hôpital Antoine-Béclère, Clamart, France; 4 Institute for Research in Biomedicine, Bellinzona, Switzerland; 5 Department of Immunology and Rheumatology, Instituto Nacional de Ciencias Mèdicas y Nutriciòn Salvador Zubiràn, Mexico City, Mexico; 6 Service de Microbiologie-Immunologie Biologique, AP-HP, Hôpital Antoine-Béclère, Clamart, France

**Keywords:** Autoimmunity, Systemic Lupus Erythematosus, B cells, Chemokines, CXCR4, CXCR7, Migration

## Abstract

**Background:**

Systemic Lupus Erythematosus (SLE) is a chronic autoimmune disease characterized by B-cell hyper-reactivity and the production of pathogenic anti-nuclear-directed auto-antibodies (Abs). B-cell ontogeny is partly dependent on the CXCL12/CXCR4 axis for which the contribution to SLE pathogenesis remains unclear. CXCR7, the novel receptor for CXCL12, is differentially expressed among memory B-cell subsets. However, its biological role in SLE remains to be explored.

**Methods:**

Relative *CXCR4* and *CXCR7* expression levels were compared by quantitative PCR in leukocytes from blood samples of 41 Mexican Mestizos patients with SLE and 45 ethnicity-matched healthy subjects. Intracellular and membrane expression of both receptors was analyzed by flow cytometry in naive and Ab-secreting B cells. B-cell responsiveness to CXCL12 was investigated using Transwell-based chemotaxis assays. Data were analyzed using the Kruskal-Wallis test for comparisons of values amongst healthy controls and patients with inactive or active SLE, and non-parametrically using the Mann–Whitney *U*-test for multiple comparisons and unpaired samples. Correlations were determined by Spearman’s ranking.

**Result:**

SLE leukocytes displayed reduced levels of *CXCR4* and *CXCR7* transcripts. In SLE patients, a significant defect in CXCR4 expression was detected at the surface of naive and Ab-secreting B cells, associated with an abnormal intracellular localization of the receptor. CXCR7 predominantly localized in cytosolic compartments of B cells from healthy and SLE individuals. Disease activity did not impact on these expression patterns. Altered receptor compartmentalization correlated with an impaired CXCL12-promoted migration of SLE B cells.

**Conclusions:**

Our data highlight a down-regulation of CXCL12 receptors on circulating B cells from SLE patients that likely influences their migratory behavior and distribution.

## Background

Systemic Lupus Erythematosus (SLE) is a prototypical chronic and systemic autoimmune disease with heterogeneous clinical manifestations and various immune dysfunctions [1]. Genetic factors play an important role in the susceptibility to SLE development [2]. Such complex disease affects multiple organs and results in death particularly when kidney damage is severe. SLE is notably characterized by a strong humoral response. A wide array of intrinsic B-cell defects, including B-cell hyper-reactivity and the production of auto-antibodies (Abs) against dsDNA and ribonucleoproteins, has been documented in SLE patients [3,4]. These pathogenic anti-nuclear Abs are thought to agglomerate and form immune complexes, thus leading to organ destruction [5]. Perturbations of peripheral B-cell homeostasis (*e.g.* increased CD27^high^ plasma cells [PC]), deposition of immune complexes and complement activation further underscore the contribution of B cells to SLE pathogenesis [6-8]. Hence, B-cell targeted therapies such as rituximab and epratuzumab, two humanized monoclonal Abs (mAbs) directed against surface molecules CD20 and CD22 respectively, are of interest for improving current treatments mostly based on non-specific immunosuppressive drugs [7,9].

Chemokines are small cytokine-like secreted proteins that govern migration of leukocytes to specific niches in lymphoid organs and inflammatory sites. They mediate their functions by binding and activating chemokine receptors, which belong to the seven-transmembrane G protein-coupled receptor family [10]. The chemokine system influences the inflammatory development and progression of B-cell mediated autoimmune diseases including SLE [3,11,12]. In this study, we focused on the CXC α-chemokine Stromal cell Derived Factor-1 (SDF-1)/CXCL12, which together with its main receptor CXCR4, constitutes the chemokine/receptor axis attracting the greatest level of interest in autoimmunity [13]. Many leukocytes, including B cells, express CXCR4 and CXCL12 is constitutively produced by stromal, epithelial, and endothelial cells notably in lymphoid organs [14,15]. Given its ability to regulate B-cell ontogeny, differentiation and homing, recent attention has been directed to the CXCL12/CXCR4 axis in SLE pathophysiology. CXCL12 is upregulated in the tubules and glomeruli of nephritic kidneys from multiple lupus-prone mouse models (*e.g.* NZB/W, BXSB, MRL.lpr) and SLE patients as well [13,16,17]. Similarly, CXCR4 hyper-expression or -activity has been reported in various leukocyte subsets of lupus mouse models [13]. Thus, in concert with CXCL12, CXCR4 could be pivotal for leukocyte trafficking into damaged kidneys from mice with lupus nephritis. In SLE patients, the situation is somewhat puzzling, as contradictory results have been reported for CXCR4 expression on peripheral blood lymphocytes. Indeed, some studies have documented down- or up-regulation of CXCR4 expression on SLE B cells, while others failed to detect any change in membrane CXCR4 expression [8,13,16,18]. To our knowledge, there is currently no consensus on CXCR4 expression and its correlation with SLE.

Little if anything is known about the expression and role of CXCR7, the second receptor for CXCL12, in SLE pathogenesis. CXCR7 binds with high affinity to CXCL12 and with lower affinity to Interferon-inducible T-cell Alpha Chemoattractant (I-TAC)/CXCL11, but its signaling capacities are still subject of debate [19,20]. Unlike CXCR4, CXCR7 does not signal through the canonical Gαi-protein pathways and thus, is unable to activate conventional signaling events [20]. However, CXCR7 could act as a decoy receptor that is able to clear CXCL12 from the surrounding medium [21-24]. CXCR7 is expressed on multiple cell types including endothelial and tumor cells and was shown to promote cell survival, adhesion and tumor growth, suggesting a signaling potential for this atypical receptor [19]. This may rely on its reported ability to behave as an endogenous β-arrestin-biased receptor or to complex with CXCR4, forming heterodimers that regulate CXCL12 responsiveness [25-28]. The expression profile of CXCR7 in mouse and human hematopoietic cells was recently disputed [19,29-31]. It appears to be expressed on B cells, tightly regulated during B-cell differentiation and correlated with the capacity of memory B cells to differentiate into Ab-secreting cells, *i.e.* PC, which are increased in the blood of patient with active SLE [6-8,21,30,32,33]. Whether CXCR7 dysregulation characterizes SLE remains to be delineated.

The aim of this study was to examine the expression of CXCR4 and CXCR7, together with CXCL12 responsiveness, in circulating B-cell subsets from Mexican Mestizos SLE patients and age- and ethnicity-matched healthy subjects. Our data reveal that both CXCR4 and CXCR7 are downregulated on SLE B cells.

## Methods

### Ethics statement

The study was carried out in accordance with good clinical practice guidelines, national laws and the Declaration of Helsinki, and was approved by the Institutional Committee of Biomedical Research (Comité Institucional de Investigación Biomédica en Humanos). All patients signed an informed consent.

### Patients, sample processing and cell culture

The study included 41 SLE patients (37 women and 4 men) with median age of 36 years (range: 21 to 62) and median disease duration of 8 years (range: 1 to 27) (Table 1). All patients were Mexican Mestizos [34], recruited in the Department of Immunology and Rheumatology of the Hospital “Instituto Nacional de Ciencias Mèdicas y Nutriciòn Salvador Zubiràn” (Mexico City, Mexico) and fulfilled at least four 1982 American Rheumatism Association revised criteria for SLE [35]. Clinical disease activity was scored using the SLE Disease Activity Index or SLEDAI [36]. 17 patients had inactive disease (SLEDAI ≤ 3) and 24 patients with indices above 3 were considered as having active disease as recently reported [37]. The five patients who had neuropsychiatric (NP) or central nervous system (CNS) history were inactive regarding these manifestations at the time of the study. All patients with renal involvement and SLEDAI > 4 had active disease at the time of the study, meaning that most of active patients displayed active renal involvement (n=20). Donors of control leukocytes consisted in age- (median 41.4 years, range: 23 to 60 years) and sex-biased (male/female ratio = 0.6) 45 healthy adult volunteers, among which 13 were referred at the Établissement Français du Sang (Rungis, France) and 32 at the Hospital “Instituto Nacional de Ciencias Mèdicas y Nutriciòn Salvador Zubiràn”. Healthy subjects were seronegative for human immunodeficiency virus, hepatitis B or C virus, without evidence of cancer, congenital heart disease or connective tissue disorder. No differences were detected in CXCL12 receptors expression and function in circulating lymphocytes from both French and Mexican healthy controls.

**Table 1:** Demographic, pathophysiological and treatment characterization of SLE patients

**Parameters**	**Inactive SLE (SLEDAI ≤ 3)**	**Active SLE (SLEDAI > 3)**	***P*value**^***a***^
**Total numbers (n)**	17	24	N/A^*b*^
**Age at study (years)**			
Median	35	36	0.474
Range	21-62	25-50	
**Sex (n)**			
Female	15	22	N/A
Male	2	2	N/A
**Duration of disease (years)**			
Median	8	8	0.577
Range	1-22	1-27	
**Clinical parameters (mean ± SD)**			
White Blood Cells (x10^9^/L)	5.6 ± 1.8	4.9 ± 1.8	0.218
**Clinical signs (n)**			
Muco-cutaneous	11	13	N/A
Haematological	8	12	N/A
Neurological/Psychiatric	-	5	N/A
Serositis	-	6	N/A
Renal	6	20	N/A
Joint	12	14	N/A
**SLE activity (mean ± SD [range])**			
SLEDAI score	0.4 ± 0.2 [0-2]	9.1 ± 3.8 [4-18]***	<0.0001
**B-cell subsets (mean ± SEM [range], x10**^**6**^**/L)**			
Total CD19^+^ B cells	554.2 ± 121.9 [54-2077]	319.3 ± 73.9 [6-1303]	0.0585
CD27^-^ naive B cells	398.6 ± 104.1 [17.9-1757]	217.1 ± 62.1 [0.7-1205]*	0.0403
Overall CD27^+^ B cells	146.2 ± 22.7 [35.1-323.2]	96.6 ± 16.8 [5.3-292.8]	0.0585
CD27^+^ memory B cells	127.5 ± 20.1 [30.3-273.6]	82.8 ± 15.2 [1.9-279.3]	0.0534
CD27^high^ plasma B cells	19 ± 3.3 [4.9-49.6]	13.9 ± 2.6 [0.9-44.2]	0.0879
**Treatment (n)**			
None	4	1	N/A
Prednisolone	5	20	N/A
Azathioprine	8	19	N/A
Methotrexate	2	1	N/A
Hydroxychloroquine	6	7	N/A
Mycophenolate Mofetil	2	3	N/A
Cyclophosphamide	-	2	N/A
Leflunomide	-	1	N/A

^*a*^Mann-Whitney *U*-test; bN/A^*b*^: Not Applicable.

Within 2 hours after blood sampling either in Mexico or France, peripheral blood mononuclear cells (PBMC) were isolated in a similar fashion from heparin-treated blood samples using Ficoll-Paque Plus (Amersham Biosciences AB, Uppsala, Sweden) density gradient centrifugation and then cryopreserved in 1 mL fetal calf serum (FCS) with 10% dimethylsulfoxide. After transportation in France, SLE and healthy PBMC were defrozen by rapid thawing at 37°C in parallel with French samples and immediately transferred to 10 mL FCS. Leukocytes were >90% viable upon thawing, as assessed by trypan-blue exclusion. After washing twice with 10 mL PBS 1X, PBMC were incubated 60 min at 37°C in RPMI medium supplemented with 20 mM HEPES and 0.5% bovine serum albumin (BSA), a step allowing resensitization of chemokine receptors including CXCR4 [38]. As specified in some experiments, whole blood and PBMC were used right after collection. Parental, *CXCR4*- and *CXCR7*-transduced HEK 293 T (HEK) cells [29] were maintained in Dulbecco’s modified Eagle’s medium supplemented with 10% FCS, glucose (4.5 g/L), 10 mM HEPES, penicillin (100 units/mL) and streptomycin (100 μg/mL) at 37°C in humidified air with 5% CO_2_.

### Real-time RT-PCR analysis

Total cellular RNA was extracted from PBMC samples using the RNeasy Plus Mini kit and genomic DNA (gDNA) Eliminator columns (Qiagen, Courtaboeuf, France) and reverse transcribed with pd(T)-15 and Moloney Murine Leukemia Virus reverse transcriptase (Fisher Bioblock, Illkirch, France). RNA quality and integrity were assessed by detecting both *18S* and *28S* ribosomal RNAs in each sample and using a BioAnalyzer (Agilent), respectively. As determined using the NanoDrop technology, all samples displayed a RNA A260/280 ratio ~ 2.0 and a RNA Integrity Number > 7 and were then processed for gene expression. Amplification of cDNAs (1 μg) was performed by quantitative real-time PCR reactions on a Light Cycler instrument (LC480, Roche Diagnostics, Meylan, France) with the LightCycler 480 SYBR Green detection kit (Roche Diagnostics) using forward (450-467) 5’-GACCGCTACCTGGCCATC-3’ and reverse (743-761) 5’-GGCAGCCAACAGGCGAAGA-3’ primers for *CXCR4* (311 bp), forward (8-29) 5’-TGCATCTCTTCGACTACTCAGA-3’ and reverse (94-113) 5’-GGCATGTTGGGACACATCAC-3’ primers for *CXCR7* (103 bp), and forward (214-223) 5’-GGGTCAGAAGGATTCCTATG-3’ and reverse (432-451) 5’-GGTCTCAAACATGATCTGGG-3’ primers for *β-actin* (237 bp). We used the LightCycler 480 Real-Time PCR system (Roche Diagnostics) with the following amplification scheme: 95°C 10 min and 40 cycles: 95°C 20 s, 60°C 15 s, 72°C 20 s. The dissociation curve method was applied according to manufacturer’s protocol (60°C to 95°C) to ensure the presence of a single specific PCR product. Relative quantification was performed with the standard curve method as we previously used [33,39]. For each tested gene (*i.e. CXCR4*, *CXCR7* or *β-actin*), standard cDNAs from PHA-stimulated PBMC were amplified along with sample cDNAs in the same PCR run. Standard curves were generated by the LC480 software (Roche Diagnostics). The threshold fluorescence common for all compared samples was set into the exponential phase of the amplifications. The target mRNA quantity in each sample was determined from the relative standard curve (using sample Ct values) and expressed in arbitrary units (AU) corresponding to the dilution factors of the standard RNA preparation. Amplification efficiency representative for each gene was determined using equation of the standard curve as reported elsewhere [40]. Results were expressed as *CXCR4*/*β-actin* on *CXCR7/β-actin* AU ratios. Internal positive controls, consisting in *CXCR4*- or *CXCR7*-transduced HEK cells, were run in parallel. We controlled that no gDNA was amplified in all cDNA samples tested using the following intron-spanning primers: forward (939-958) 5’-CACTCCCGCCCAATATACCC-3’ and reverse (1218-1237) 5’-ACACACAAAGAGGCCACTCC-3’ primers for *CXCR4* (298 bp) and forward (353-372) 5’-CTACAGCCACAGAAAGCGGA-3’ and reverse (527-546) 5’-GGAAAGGAAAACTGCCAGCG-3’ primers for *CXCR7* (193 bp).

### Flow-cytometric analysis

**Figure 1:**
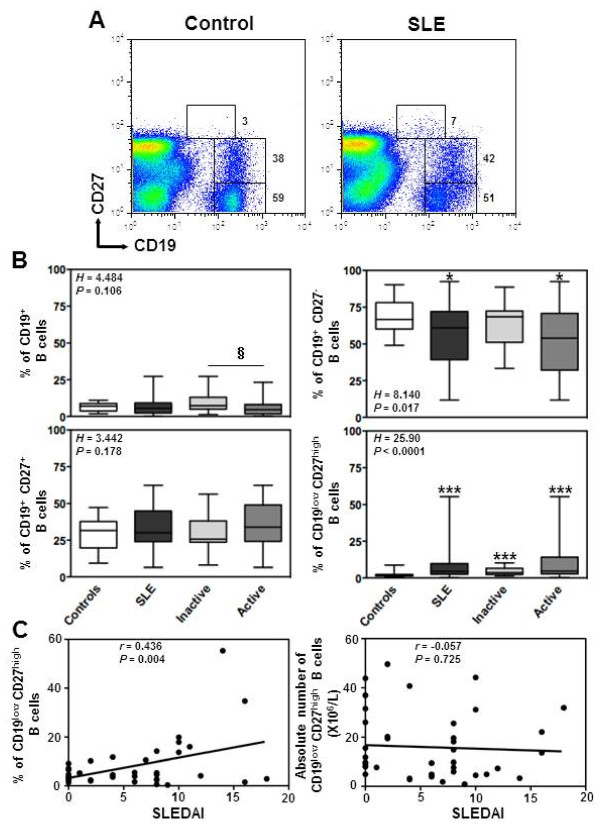
**Increased frequency of circulating plasma cells in SLE patients. (A)** Double staining with CD19 and CD27 was performed on PBMC to delineate naive (CD19^+^CD27^-^) B cells, memory (CD19^+^CD27^+^) B cells and plasma (PC, CD19^low^CD27^high^) cells. Thresholds and fluorescence gates used for the statistical evaluation of CD27^-^, CD27^+^ and CD27^high^ B lymphocytes are indicated as well as the corresponding frequency of these subsets among total B cells. Expression of CD27 on CD19^+^ peripheral B cells is shown for a representative healthy blood donor and a patient with active SLE. **(B)** Comparison of the frequencies of total B cells among PBMC and of the different B-cell subpopulations from SLE patients (n = 41), distributed according to disease activity, *i.e.* inactive (n = 17) versus active (n = 24), and from healthy individuals (n = 45). The proportions were determined by flow-cytometric analysis as shown in **A**. Results are depicted as box plots, with median (horizontal line within each box) and 10^th^, 25^th^, 75^th^, and 90^th^ percentiles (bottom bar, bottom of box, top of box, and top bar, respectively). Kruskal-Wallis *H* test and associated *P* values are indicated. *P < .05 and ***P < .0005 compared with control B cells. ^§^*P* < .05 compared with B cells from patients with inactive SLE (as determined using the Mann–Whitney *U*-test). **(C)** Frequency (left panel) or total number (right panel) values obtained within CD27^high^ PC for each SLE patient were plotted as a function of SLEDAI scores. Correlation factor (*r*, Spearman’s) and *P* value are indicated.

The frequency of each B-cell subset and membrane expression levels of both CXCR4 and CXCR7 were determined by four-color flow-cytometric analysis. For this purpose, PBMC were incubated with the following mAbs (unless specified from BD Pharmingen, Le Pont de Claix, France): fluorescein isothiocyanate (FITC)-conjugated mouse anti-human CD27 (clone M-T271), unconjugated mouse anti-human CXCR7 (clone 9C4) followed by a phycoerythrin (PE)-conjugated goat anti-mouse F(ab’)_2_ Ab (Dako, Trappes, France) as previously described [29], PE-conjugated mouse anti-human CD38 (clone HIT2) or CXCR7 (clone 11G8, R&D Systems, Lille, France or clone 8F11-M16, BioLegend, San Diego, CA), peridinin chlorophyll protein-Cy5 (PerCP-Cy5)-conjugated mouse anti-human CD19 (clone HIB19), and allophycocyanin (APC)-conjugated mouse anti-human CXCR4 (clone 12G5) or CD20 (clone 2H7). Isotype- and species-matched Abs were used as negative controls. After wash in PBS 1X, cells were analyzed on a FACSCalibur cytometer (BD Biosciences) using the CellQuest software (BD Biosciences). At least 30,000 events were collected for each analysis after gating on forward and side scatter to select living lymphocytes. The frequencies of naive (CD19^+^CD27^-^), memory (CD19^+^CD27^+^) and plasma (CD19^low^CD27^high^) B cells were calculated according to statistical threshold sets in reference of control staining (Figure 1A and Table 1), as previously reported [6]. *CXCR4*- or *CXCR7*-transduced HEK cells were used as positive controls for specific staining. To assess total pool of CXCR4 and CXCR7, *i.e.* membrane and intracellular detection, PBMC were incubated with FITC-conjugated mouse anti-human CD27 and PerCP-Cy5-conjugated mouse anti-human CD19 mAbs, washed twice in PBS 1X, fixed and permeabilized using the BD Cytofix/Cytoperm Fixation/Permeabilization Kit (BD Biosciences) according to the manufacturer’s instructions. Cells were subsequently stained with 9C4 and 12G5 mAbs, or the corresponding isotype Ab, at room temperature for 30 min and then analyzed by flow cytometry.

### Chemotaxis assays

Chemotaxis was performed using a Transwell assay upon CXCL12 or EBI1-Ligand Chemokine (ELC)/CCL19 induction as previously described [29]. Briefly, 3 × 10^5^ PBMC in 150 μL RPMI medium supplemented with 20 mM HEPES and 0.5% BSA were added to the upper chamber of a 6.5-mm diameter, 5-μm pore polycarbonate Transwell culture insert (Corning costar, Brumath, France). The same media (600 μL) with or without CXCL12 (chemically synthesized by Dr. F. Baleux, Unité de Chimie Organique, Institut Pasteur, Paris, France) or CCL19 (R&D Systems) diluted at various concentrations was placed in the lower chamber. AMD3100 (Sigma-Aldrich, Saint-Quentin Fallavier, France) was used at 1 μM to inhibit CXCR4-dependent signaling. Input leukocytes that migrated to the lower chamber after 2-hour incubation at 37°C in humidified air with 5% CO_2_ were collected, stained with CD19, CD20, CD38 and CD27 mAbs, and counted by flow cytometry. The fraction of cells migrating across the polycarbonate membrane was calculated as follows: [(number of cells migrating to the lower chamber in response to chemokine or medium)/(number of cells added to the upper chamber at the start of the assay)] × 100.

### Statistical analysis

Unless specified, all values are expressed as median (25^th^ and 75^th^ percentiles). Data were analyzed using the Kruskal-Wallis test for comparisons of values amongst at least three groups, *i.e.* healthy controls, patients with inactive or active SLE, when required, and non-parametrically using the Mann–Whitney *U*-test (Prism software, GraphPad, La Jolla, CA) for multiple comparisons and unpaired samples. Correlations were determined by Spearman’s ranking. *P* values lower than 0.05 were considered statistically significant.

## Results

### Altered B-cell distribution in the peripheral blood of SLE patients

Cryopreserved PBMC of all 41 Mexican Mestizos patients with SLE and 45 age- and ethnicity-matched independent healthy subjects were analyzed by flow cytometry to determine the frequencies of naive, memory and plasma B cells. Consistent with previous works [1,41], several abnormalities of the B-cell compartment were detected in the peripheral blood of SLE patients including a B-cell lymphopenia that predominantly affects naive B cells (Figure 1B and Table 1). In contrast, a significant increase in the frequency of PC was observed in patients with SLE (Figure 1A). Comparison of the proportions of the different B-cell subpopulations from SLE patients distributed according to disease activity, *i.e.* inactive (n = 17, SLEDAI ≤ 3) versus active (n = 24, SLEDAI > 3), revealed more marked B-cell alterations in the blood of patients with active SLE. No significant correlation could be detected between white blood cell count and disease activity, duration or organ alteration (*i.e.* renal, muco-cutaneous). In accordance with previous reports [6,32], only the frequency of PC positively and significantly correlated with the disease activity among peripheral B-cell subsets (Figure 1C). Taken together, our data confirmed perturbations of peripheral B-cell homeostasis, which were more pronounced in patients with active disease. The implication of CXCR4 and CXCR7 in the terminal differentiation of B cells into Ab-secreting cells [15,30] prompted us to investigate whether altered B-cell distribution in SLE was associated with changes in CXCL12 receptors expression.

### Reduced levels of *CXCR4* and *CXCR7* mRNAs in SLE leukocytes

**Figure 2:**
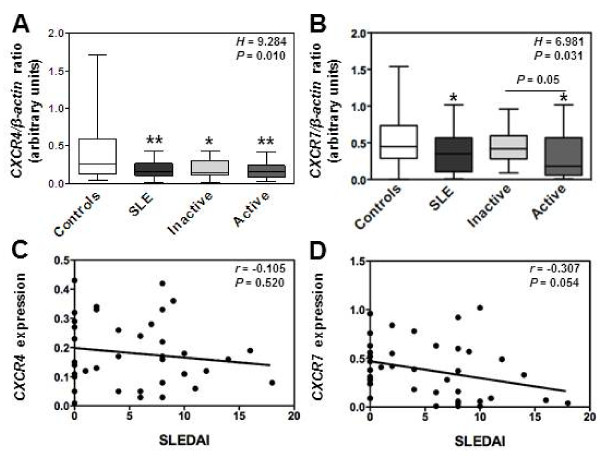
**Decreased levels of *CXCR4 *and *CXCR7 *mRNAs in active SLE leukocytes. (A and B)** The relative levels of *CXCR4 ***(A)** and *CXCR7 ***(B)** transcripts in PBMC from SLE patients, distributed according to disease activity (*i.e.* inactive versus active), were compared by real-time PCR with those from healthy individuals. Each individual sample has been run in triplicate. Results are expressed as *CXCR4*/*β-actin* or *CXCR7*/*β-actin* AU ratio and presented as box plots with median values, 25^th^ and 75^th^ quartile and the range of values. Kruskal-Wallis *H* test and associated *P* values are indicated. *P < .05 and **P < .005 compared with control leukocytes (as determined using the Mann–Whitney *U*-test). **(C and D)** Correlation scatter plots of *CXCR4***(C)** or *CXCR7***(D)** mRNA levels and SLEDAI scores. Correlation factor (*r*, Spearman’s) and *P* value are indicated.

We have set up real-time PCR analysis to quantify relative levels of *CXCR4* and *CXCR7* mRNAs in SLE PBMC as compared with those detected in the 45 healthy individuals. Transcripts encoding CXCR4 were readily detectable in leukocytes from healthy subjects (Figure 2A). Likewise, *CXCR7* mRNAs could be amplified in control leukocytes (Figure 2B). Expression levels of both *CXCR4* and *CXCR7* mRNAs scattered over a large range among donors, likely as the consequence of an inter-individual variability [33,42,43]. However, the levels of *CXCR4* and *CXCR7* transcripts were significantly decreased in leukocytes from patients with active disease as compared to control cells (Figures 2A and B). Amounts of *CXCR4* and *CXCR7* mRNAs seemed to negatively correlate with SLEDAI scores, although this did not reach statistical significance (Figures 2C and D). Disease duration and muco-cutaneous manifestation did not impact on *CXCR4* or *CXCR7* expression. When comparing the group with renal failure history (n=26) with that of non-renal-affected patients (n=15), we did not find any significant differences between both groups regarding *CXCR4* or *CXCR7* expression. Thus, our results unveiled a decrease in *CXCR4* and *CXCR7* mRNA content in PBMC from SLE patients, which is more pronounced in those with active disease.

### Defective membrane expression of CXCR4 and CXCR7 in SLE B lymphocytes

**Figure 3:**
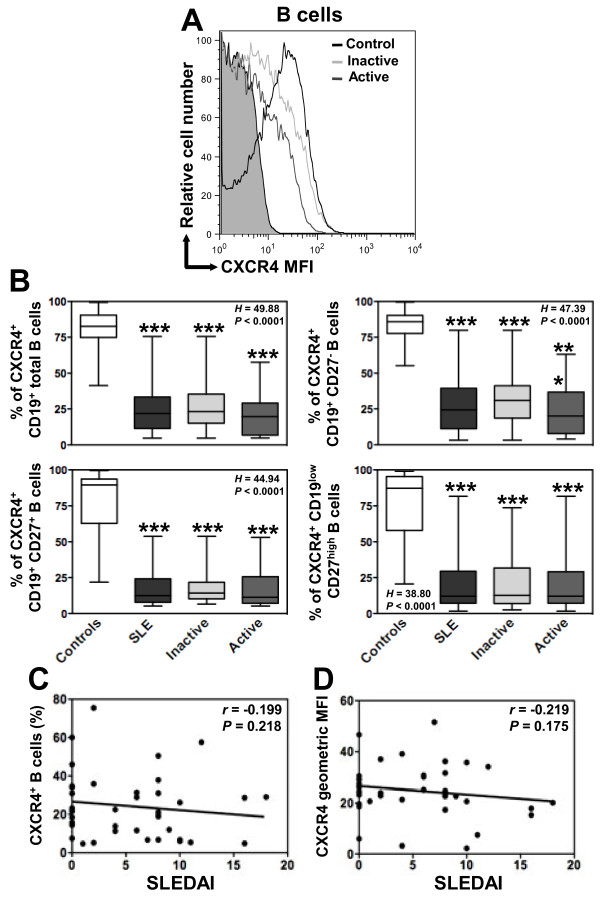
**Loss of CXCR4 expression on SLE B cells. (A)** Membrane expression of CXCR4 on CD19^+^-gated PBMC from healthy and SLE subjects was determined by flow cytometry using the APC-conjugated 12G5 (empty histograms) or isotype control (filled histogram) mAb. Displayed data are representative plots of the mean fluorescence intensity (MFI) of CXCR4 at the surface of total B cells from a healthy individual and two patients with either inactive or active SLE. **(B)** The percentage of total (CD19^+^) B cells, naive (CD19^+^CD27^-^) B cells, memory (CD19^+^CD27^+^) B cells and PC (CD19^low^CD27^high^) expressing CXCR4 in healthy and SLE subjects are given. Box plots show the median values, 25^th^ and 75^th^ quartile and the range of values. Kruskal-Wallis *H* test and associated *P* values are indicated. ***P < .0005 compared with control B cells (as determined using the Mann–Whitney *U*-test). **(C and D)** CXCR4-positive fraction **(C)** or MFI **(D)** values obtained within total B cells for each SLE patient were plotted as a function of SLEDAI scores. Correlation factor (*r*, Spearman’s) and *P* value are indicated.

We next assessed whether such transcriptional anomaly could result in reduced protein levels on circulating B cells. A global flow-cytometric analysis of the expression of CXCR4 and CXCR7 was performed on the three B-cell subpopulations, *i.e.* naive B cells, memory B cells and PC, in the control and SLE groups. As shown in Figure 3A, most peripheral blood B cells of healthy donors expressed CXCR4 (median value 82%, range: 41 to 99%). The percentage of CXCR4-positive cells decreases as control B cells differentiate to PC (Figure 3B). In addition, CXCR4 surface expression reached different levels on total B cells (geometric mean fluorescence intensity (MFI) median value 55, range: 11 to 217) and the different B-cell subsets as well (Additional file 1: Figure S1A). In line with previous works [41], CXCR4 expression on circulating B cells was significantly lower in SLE patients than in healthy controls (Figures 3A and B and Additional file 1: Figure S1A). Because the decrease in CXCR4 expression could be due to changes in the proportion of B-cell subsets or variations in expression levels within subsets, we performed an intra-subset analysis. Both frequencies of CXCR4-positive B cells and expression levels of CXCR4 within naive, memory and PC subsets were markedly reduced in patients with SLE (Figure 3B and Additional file 1: Figure S1A). Strikingly, B cells from patients with either inactive or active disease displayed a comparable defect in cell surface CXCR4 expression in the three subsets analyzed. Therefore, and similarly to the disease duration or organ manifestation (*i.e.* renal, muco-cutaneous), the disease activity did not impact on the level of CXCR4 expression. Consequently, no significant correlation could be detected between SLEDAI scores and CXCR4 expression on total B cells (Figures 3C and D).

**Figure 4:**
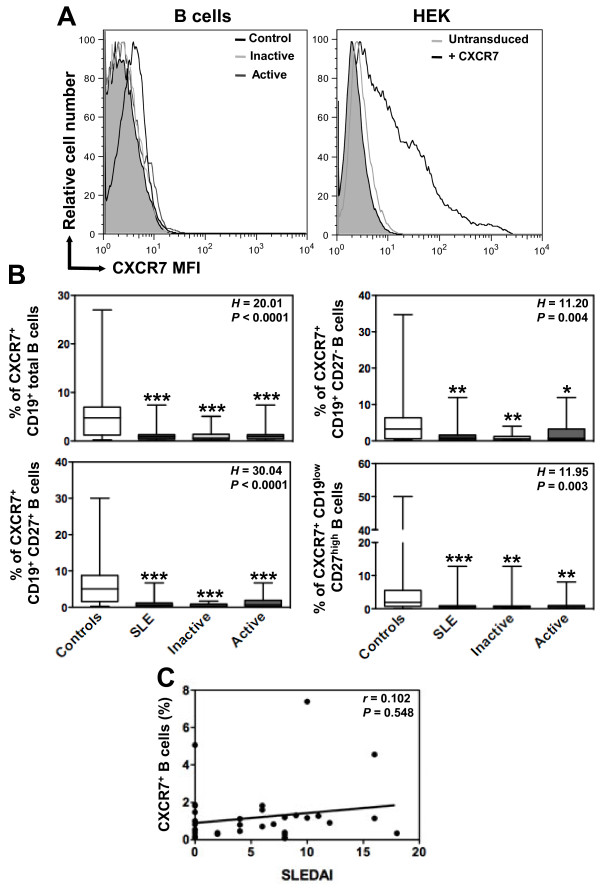
**Altered CXCR7 expression on SLE B cells. (A)** Surface expression of CXCR7 on CD19^+^-gated PBMC from healthy and SLE subjects was determined by flow cytometry using the unconjugated 9C4 (empty histograms) or isotype control (filled histograms) mAb followed by a PE-conjugated goat anti-mouse F(ab’)_2_ Ab. *CXCR7*-transduced (*versus* parental) HEK 293 T (HEK) cells were used as a positive control for staining. Displayed data are representative plots of the MFI of CXCR7 at the surface of total B cells from a healthy individual and two patients with either inactive or active SLE (left panel) or of HEK cells transduced with *CXCR7* or left untransduced (right panel). **(B)** The percentage of total (CD19^+^) B cells, naive (CD19^+^CD27^-^) B cells, memory (CD19^+^CD27^+^) B cells and PC (CD19^low^CD27^high^) expressing CXCR7 in healthy and SLE subjects are shown. Box plots show the median values, 25^th^ and 75^th^ quartile and the range of values. Kruskal-Wallis *H* test and associated *P* values are indicated. *P < .05, **P < .005 and ***P < .0005 compared with control B cells (as determined using the Mann–Whitney *U*-test). **(C)** CXCR7-positive fraction values obtained within total B cells for each SLE patient were plotted as a function of SLEDAI scores. Correlation factor (*r*, Spearman’s) and *P* value are indicated.

We concomitantly assessed CXCR7 protein expression on B-cell subpopulations using the 9C4 mAb, which has been previously reported to specifically react with human CXCR7 [29,30,33]. Confirming this, the 9C4 mAb stained *CXCR7*-transduced, but not parental, HEK cells (Figure 4A). Compared with CXCR4, we observed only low levels of CXCR7 (median value 4.8%, range: 0.2 to 27%) at the surface of CD19^+^-gated PBMC from healthy individuals (Figure 4B). CXCR7 remained scarcely expressed on maturating, including memory, B cells. Similar staining patterns were obtained using commercially available 11G8 and 8F11-M16 anti-CXCR7 mAbs. CXCR7 expression on naive and overall CD27^+^ B cells was still significantly lower in SLE patients than in healthy controls. However, as for CXCR4, there was no correlation between CXCR7 expression levels and the clinical activity of SLE (Figure 4C).

When we compared patients with (n=25) or without (n=16) prednisolone-based treatment, no significant differences were found between both groups in term of receptors levels. As most active patients received 30 or 50 mg/day prednisolone, we thus cannot assigned an impact of steroids on CXCR4 or CXCR7 expression. Taken together, these findings unraveled a down-regulation of CXCL12 receptors on circulating B-cell subsets from SLE patients. We next addressed whether this loss of membrane CXCR4 and CXCR7 expression was associated with an abnormal intracellular compartmentalization of the receptors.

### Intracellular localization of CXCL12 receptors in SLE B lymphocytes

**Figure 5:**
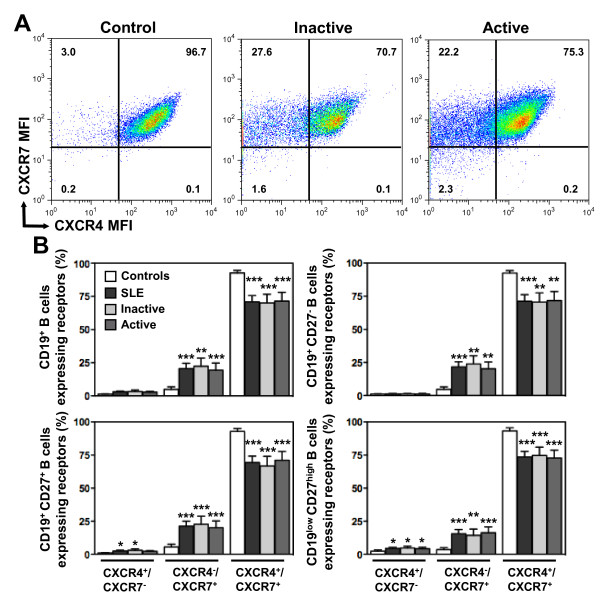
**Intracellular localization of CXCR4 and CXCR7 in SLE B cells. (A)** Total pools of CXCR4 and CXCR7 were simultaneously detected by flow-cytometric analysis in CD19^+^-gated PBMC from healthy and SLE subjects. Staining was performed on fixed and permeabilized leukocytes with both 9C4 (PE) and 12G5 (APC) mAbs. Quadrants were set on controls stained with the corresponding isotype control. Representative dot-plots demonstrating coexpression of CXCR4 and CXCR7 in total B cells from a healthy individual and two patients with either inactive or active SLE are shown. **(B)** The proportions (mean ± SEM) of total (CD19^+^) B cells, naive (CD19^+^CD27^-^) B cells, memory (CD19^+^CD27^+^) B cells and PC (CD19^low^CD27^high^) expressing CXCR4 and/or CXCR7 in healthy and SLE subjects are given. *P < .05, **P < .005 and ***P < .0005 compared with control B cells.

CXCR4 is mostly expressed at the surface of circulating leukocytes from healthy donors, while its intracellular pool is rather minor [44,45]. By contrast, CXCR7 is rarely, or even not, expressed at the membrane of human and mouse leukocytes [21,29,30,46]. Some studies indicated that CXCR7 is predominantly localized underneath the plasma membrane [47], while others failed to detect it intracellularly [31]. Stimulated by this puzzling picture of the intracellular detection of CXCR4 and CXCR7, we assessed by flow cytometry the expression of both receptors in CD19^+^-gated PBMC after permeabilization. This step allowed detecting relative total, *i.e.* both cell surface and intracellular, pools of CXCR4 and CXCR7 in B cells. As shown in Figure 5A, the majority of total B cells from healthy individuals coexpressed CXCR4 and CXCR7 (median value 97%, range: 51.9 to 99%). About 1% and 3% of B cells only expressed CXCR4 or CXCR7, respectively, whereas roughly < 1% of the cells expressed neither CXCR4 nor CXCR7. A similar pattern was found in the different B-cell subsets (Figure 5B). These findings, combined with those displayed in Figures 3 and 4, confirmed the differential cellular compartmentalization of both CXCR4 and CXCR7, *i.e.* predominant cell surface detection of CXCR4 (>80% total pool) and intracellular localization of CXCR7 (>90% total pool).

The situation was somewhat different in permeabilized PBMC from SLE patients. The fraction of total B cells coexpressing both receptors was slightly decreased by ~20-30% relative to control cells, while the proportion of B cells expressing only CXCR4 or CXCR7 was significantly increased (Figure 5). The defect in total CXCR4 expression was comparable among naive, memory and PC subsets from patients with either inactive or active disease. These data further revealed a significant intracellular detection of both CXCR4 (>60% total pool) and CXCR7 (>90% total pool) in SLE B cells. In line with this, geometric MFI values for total pools of CXCR4 and CXCR7 were roughly comparable in B cells from healthy and SLE subjects, while membrane expression levels of CXCR4 were decreased on SLE B cells (Additional file 1: Figure S1). Collectively, our findings established that naive and overall CD27^+^ B cells from both healthy and SLE individuals displayed a nearly exclusive expression of CXCR7 in cytosolic compartments. The loss of membrane CXCR4 expression in SLE B cells was accompanied by its abnormal intracellular localization.

### Impaired CXCL12-promoted chemotaxis of SLE B lymphocytes

**Figure 6:**
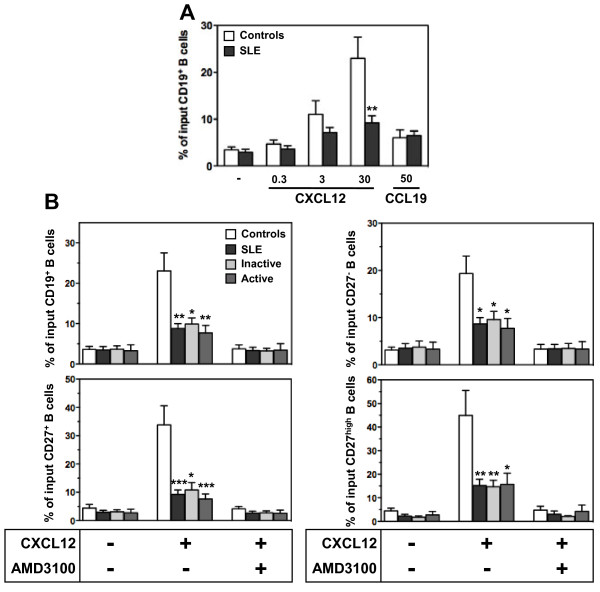
**Loss of CXCL12 responsiveness in SLE B cells. (A and B)** PBMC from independent healthy individuals (n = 9) and patients (SLE, n = 14), with either inactive (n = 7) or active (n = 7) disease, were tested for their ability to migrate in response to the indicated concentration **(A)** of chemokine or 30 nM of CXCL12 **(B)**. Transmigrated cells recovered in the lower chamber were stained with mAbs specific for CD19, CD20, CD38 and CD27 antigens and counted by flow cytometry. Inhibition of cell migration by AMD3100 added in both chambers is shown. Results (mean ± SEM) are from 4 independent experiments and expressed as the percentage of input total (gated CD19^+^) B cells (A and B, upper left), naive (gated CD19^+^CD20^+^CD27^-^CD38^-^) B cells (B, upper right), memory (gated CD19^+^CD20^+^CD27^+^CD38^-^) B cells (B, lower left) or PC (gated CD19^low^CD20^low^CD27^high^CD38^high^) (B, lower right) that migrated to the lower chamber. *P < .05, **P < .005 and ***P < .0005 compared with control B cells.

We thus questioned whether impaired compartmentalization of CXCL12 receptors, *i.e.* decreased at the membrane and increased at the intracellular level, in SLE B cells impacted on CXCL12 responsiveness. We investigated the migratory response of CD19^+^-gated PBMC from healthy and SLE subjects to CXCL12 using a Transwell-based chemotaxis assay. Addition of CXCL12 resulted in a dose-dependent chemotactic response of both control and SLE B cells (Figure 6A). However, SLE B cells displayed weaker migratory responses to all concentrations tested, indicating a lower efficiency of chemotaxis to CXCL12. In contrast, CCR7-dependent migration following CCL19 stimulation was observed to be in the same range for SLE and control B cells. We then compared chemotactic responses of the different B-cell subsets from patients with either inactive or active SLE to those obtained with control cells. The CXCL12 dose was chosen so that migration was detectable for B cells harboring low CXCL12 receptors expression (*i.e.* SLE group) without reaching saturating concentrations for B cells with higher receptor expression (*i.e.* control group). Upon exposure to 30 nM CXCL12, the percentage of specific migration of B cells from healthy donors was of ~15%, ~30% and ~40% in the naive, memory and PC subsets, respectively (Figure 6B). Remarkably, circulating B cells from patients with inactive or active disease shared an impaired CXCL12-promoted chemotaxis, with a significant reduction (> 60% as compared to control cells) of specific migration in the three subsets analyzed. Such decrease in migration efficiency was consistent with the severe decrease in CXCR4 expression observed on all SLE B-cell subsets. The residual migration of SLE B cells upon CXCL12 exposure was totally inhibited by the specific CXCR4 antagonist AMD3100 [48]. Overall, these findings suggested that the defect in membrane CXCR4 expression contributes to the loss of CXCL12 responsiveness of SLE B lymphocytes.

## Discussion

In the present study, we provided novel insights to SLE pathophysiology based on the systematic evaluation of CXCL12 receptors expression in circulating B cells from a large cohort of 41 Mexican Mestizos patients. Our findings revealed reduced levels of *CXCR4*, and to a lesser extent of *CXCR7*, transcripts in SLE PBMC compared to controls. SLE was associated with a strong defect of membrane CXCR4 expression in naive and Ab-secreting B cells accompanied by an abnormal intracellular detection of CXCR4. CXCR7 was scarcely expressed on control and SLE B cells and predominantly localized in cytosolic compartments. Altered receptor compartmentalization was associated with an impaired CXCL12-promoted migration of SLE B cells. As the majority (n=23) of active patients were treated with at least two drugs, we cannot assess the effects of each immunosuppressive therapy on CXCL12 receptors expression and function. Neither disease activity or duration nor muco-cutaneous and renal manifestations significantly influenced CXCR4 and CXCR7 protein expression. Conversely, when we applied a single cut-off at 21.79% and 0.83%, the respective median value for CXCR4- and CXCR7-positive B cells in the entire cohort, for the classification of patients displaying low or high levels of CXCL12 receptors expression, no clinical difference was found between the two groups (*P* = 0.52 and *P* = 0.21 for CXCR4 and CXCR7 respectively, Mann–Whitney *U*-test). Thus, it would be worthwhile to investigate groups of active patients with joint/cutaneous, hematological, CNS, and pure renal involvement in order to differentiate how these manifestations impact on receptors expression.

CXCR4 is involved in B-cell differentiation and homing as well as in the inflammatory development and progression of B-cell mediated autoimmune disorders including SLE [3,13]. In line with previous works [13,15,42], we observed that the vast majority of circulating total B cells from healthy donors stained for CXCR4, while the frequency of CXCR4-positive B cells decreased upon differentiation. Similarly, an inverse correlation of membrane CXCR4 expression and maturating stage of B cells was detected in SLE patients. However, the frequency of CXCR4-positive B cells within naive, memory and PC subsets was significantly decreased, suggesting that the defect in membrane CXCR4 expression results from an intrinsic CXCR4 anomaly rather than the expansion of a CXCR4-negative B-cell subpopulation. Circulating B cells from patients with either inactive or active disease displayed a comparable defect in membrane CXCR4 expression, indicating that the disease activity did not impact on the level of CXCR4 expression. Thus, our findings provided evidence that SLE B cells display a loss of membrane CXCR4 expression. In agreement with this, Henneken and coworkers previously showed a decrease in CXCR4-positive naive and overall CD27^+^ B cells from 4 SLE patients [41]. Because CXCR4 is virtually expressed on all B-cell subsets, changes in CXCL12 responsiveness that occur throughout lymphocyte ontogeny were proposed to result from differences in the activation of CXCR4-mediated signaling [15]. For instance, CXCR4 expression is downregulated upon B-cell receptor engagement [49]. It is tempting to speculate that the loss of CXCR4 expression in SLE reflects, at least in part, chronic *in vivo* B-cell activation.

While there is a relative consensus on CXCR4 anomaly in B cells of several mouse models of lupus nephritis [13,50], the situation is still unclear in humans. Indeed, Wang and coworkers observed increased CXCR4 levels in circulating B and T cells from 31 SLE patients [16]. These findings have been partially confirmed by Rodriguez-Bayona and coworkers who detected in a larger cohort composed of 69 patients a slight increase in CXCR4 expression restricted to non-switched memory B cells and sparing other B-cell subsets [8]. The reasons for discrepancies on CXCR4 profiles between our work and principally that of Wang and coworkers are unclear but may relate to differences in sample handling or ethnical origin of patients [51]. Indeed, whereas Wang and coworkers performed CXCR4 staining on whole blood [16], we analyzed CXCL12 receptors patterns on PBMC, indicating that the Ficoll-based isolation could constitute a cause for the differences on CXCR4 expression. However, and in contrast to the recent study by Nieto and coworkers [38], we found only discrete differences in CXCR4 expression at the surface of control B cells after cell isolation as compared to whole blood samples (Additional file 2: Figure S2). Cryopreservation of PBMC might also constitute a source of changes in labile phenotypic markers and B-cell subset distribution. Recently, Faint and coworkers showed that cryopreserved sample batching, and subsequent thawing for deferred analysis, did not alter the proportion of lymphocyte subsets but led to an increased CXCR4 expression on lymphocytes [52]. In our hands, the cryopreservation step indeed influenced positively CXCR4 expression on control B cells (Additional file 2: Figure S2). Thus, this process cannot solely explain the CXCR4 down-regulation we describe here. SLE represents a polygenic disease for which several clinical differences among ethnic groups have been documented [2,53,54]. Having focused on a cohort of 41 Mexican Mestizos patients [34], we differed from other studies, which have assessed CXCR4 expression on lymphocytes from patients with various ethnical origins [8,16]. Therefore, we cannot rule out that heterogeneity in the genetic background could account for contradictory results.

The molecular basis underlying the defect in CXCR4 expression on SLE B cells is unclear but might involve both impaired receptor synthesis and intracellular trafficking. We unveiled decreased *CXCR4* expression in PBMC from SLE patients. Such transcriptional anomaly, which is consistent with a recent study [18], could result in reduced protein levels in circulating leukocytes including the B-cell compartment. Whether epigenetic (*e.g.* promoter hypermethylation) or post-transcriptional (*e.g.* microRNA) regulatory mechanisms account for the decrease in the steady-state levels of *CXCR4* mRNAs will be the focus of future investigations on sorted B cells from patients. Our findings were enough indicative to delineate how such transcriptional anomaly could impact on protein levels, especially in circulating B cells. Flow-cytometric analysis revealed a significant decrease in the total pool of CXCR4 together with an impaired compartmentalization of the receptor, *i.e.* reduced at the cell surface and increased at the intracellular level, in all B-cell subsets from SLE patients irrespective of their disease activity status. The different cytokine milieu in SLE patients could alter the expression and activity of CXCR4. An increase in serum TNF-α and type I IFN levels is characteristic of SLE [11] and both cytokines are known to down-regulate CXCR4 [55,56]. The pro-inflammatory cytokine IL-17 might be involved in such phenomenon, as it increased in the serum of SLE patients [57] and down-modulated the responsiveness of germinal center B cells to Cxcl12 in autoimmune BXD2 mice [58]. Interestingly, an increase of CXCL12 in the serum of SLE patients has been reported [59]. Since CXCR4 engagement upon CXCL12 stimulation paradigmatically leads to receptor internalization, one can speculate that chronic exposure to CXCL12 is another potential explanation for abnormal CXCR4 expression on autoreactive B cells.

The study was extended to the expression of CXCR7, which is another receptor for CXCL12 but, unlike CXCR4, it has never been investigated in the context of lupus pathogenesis. Surface expression of CXCR7 is well documented on activated endothelium, fetal liver cells, neurons and multiple cancer cells [19,60]. Many groups have detected CXCR7 products in mouse and human leukocytes, although one study challenged this view [31,33]. Our real-time PCR-based analysis permitted to readily detect mRNAs encoding CXCR7 in PBMC from healthy subjects, further corroborating previous studies demonstrating unambiguously transcript amplification in mouse and human monocytes, dendritic cells, CD4^+^ T cells, neutrophils and B cells [19,30,33,61,62]. Nevertheless, SLE leukocytes displayed a slight decrease in the steady-state level of *CXCR7* transcripts. Further in-depth studies are required to determine whether transcriptional regulatory mechanisms account for heterogeneous *CXCR7* expression in SLE. Like for CXCR4, we found no differences in CXCR7 expression at the surface of control B cells after Ficoll-based cell isolation as compared to whole blood samples (Additional file 3: Figure S3). In contrast, the cryopreservation step led to a reduced CXCR7 expression on B cells. Thus, we cannot exclude that cryopreserved sample batching contributes to low levels of CXCR7 on SLE B cells.

Intracellular compartmentalization of CXCL12 receptors was simultaneously assessed by flow cytometry after cell permeabilization, and in line with previous works [20,47], unraveled that CXCR7 predominantly localized in cytosolic compartments of B-cell subsets from healthy and SLE subjects. Such peculiar intracellular pattern is consistent with the notion that CXCR7 rapidly cycles between the plasma membrane and intracellular compartments either spontaneously or upon exposure to its ligand [20,24,28,47]. The main function of endothelium-expressed CXCR7 is likely sequestration of CXCL12 as proposed in homeostatic and pathological, including autoimmune, conditions [24,46]. Additionally, mouse CXCR7 seems to function as a sink for CXCL12 and contributes to marginal zone B-cell retention as well as kidney development [22,63]. Recently, we identified CXCR7 as an active scavenger for CXCL12 on human tonsil-derived B cells [33]. Although the relevance of this activity in human B-cell homeostasis remains to be determined, one can speculate that CXCR7 acts as a decoy receptor in B cells, thereby clearing CXCL12 from the surrounding medium and controlling CXCR4 expression and/or signaling. A crosstalk between intracellular CXCR4 and CXCR7 could also be involved in the modulation of CXCL12 activities as previously proposed [47]. Finally, we cannot exclude that defective *CXCR4* and *CXCR7* expression in SLE leukocytes impacts on the anterograde trafficking, *i.e.* from synthesis in the endoplasmic reticulum to the plasma membrane, of both receptors, resulting in their altered cellular distribution [64]. Studying the relevance of CXCR7 in B-cell biology was not in the scope of this study but clearly deserves further investigations using selective antagonists for CXCR4 and CXCR7.

## Conclusions

Our findings highlight a previously unappreciated down-regulation of CXCR4 and CXCR7 on circulating B cells from SLE patients. Such anomaly is associated with intracellular localization of both receptors and a loss of CXCL12 responsiveness. This is the first study assessing simultaneously the status of CXCL12 receptors in autoimmune-biased B cells. This work offers important pathophysiological insights into the migratory behavior of autoreactive B cells from SLE patients, potentially permitting new therapeutic avenues.

## Abbreviations

Ab: Antibody; APC: Allophycocyanin; BSA: Bovine serum albumin; CNS: Central nervous system; FCS: Fetal calf serum; FITC: Fluorescein isothiocyanate; MFI: Mean fluorescence intensity; NP: Neuropsychiatric; PBMC: Peripheral blood mononuclear cells; PC: Plasma cells; PE: Phycoerythrin; PerCp-Cy5: Peridinin chlorophyll protein-Cy5; SDF-1: Stromal cell Derived Factor-1; SLE: Systemic Lupus Erythematosus; SLEDAI: Systemic Lupus Erythematosus Disease Activity Index.

## Competing interests

The authors declare that they have no competing interests.

## Authors’ contributions

Conceived and designed the experiments: VB, AB, DE and KB. Performed the experiments: VB, AB, CF and KB. Analyzed the data: VB, AB, CF and KB. Wrote the paper: VB, AB and KB. Provided funding: DE and KB. Provided experimental tools and contributed to manuscript editing: VM, MT and LL. Provided patient samples and clinical features: GL, JJ-O and LL. All authors read and approved the final manuscript.

## Supplementary Material

Additional file 1: Figure S1 Distribution of CXCR4 and CXCR7 in SLE B cells. **(A)** Expression of CXCR4 on CD19^+^-gated PBMC from SLE patients (n = 41), distributed according to disease activity, *i.e.* inactive (n = 17) versus active (n = 24), and healthy individuals (n = 45) was determined by flow-cytometric analysis (FACSCalibur, BD Biosciences) using the APC-conjugated 12G5 mAb. The geometric mean fluorescence intensity (MFI) of CXCR4 at the surface of total (CD19^+^) B cells, naive (CD19^+^CD27^-^) B cells, memory (CD19^+^CD27^+^) B cells and PC (CD19^low^CD27^high^) from healthy and SLE subjects are displayed. **(B)** Total pools of CXCR4 and CXCR7 were detected by flow cytometry by staining fixed and permeabilized leukocytes with 9C4 (PE) and 12G5 (APC) mAbs. The geometric MFI of CXCR4 or CXCR7 was evaluated in total B cells. Box plots show the median values, 25^th^ and 75^th^ quartile and the range of values. Kruskal-Wallis *H* test and associated *P* values are indicated. ***P < .0005 compared with control B cells. ^§^*P* < .05 compared with B cells from patients with inactive SLE (as determined using the Mann–Whitney *U*-test). Click here for file

Additional file 2: Figure S2 Sample processing modulates membrane CXCR4 expression. **(A)** Membrane expression of CXCR4 on control CD19^+^-gated B cells was determined by flow-cytometric (FACS Fortessa, BD Biosciences) analysis using the PE-conjugated 12G5 (empty histograms) or isotype control (filled histogram) mAb. Displayed data are representative plots of the MFI of CXCR4 at the surface of total B cells from 4 independent healthy Caucasian women (age median 35 years, range: 24 to 47 years) obtained either after staining on whole blood (Blood) or fresh (Ficoll) or cryopreserved (Cryo) PBMC. **(B)** The percentage of total (CD19^+^) B cells, naive (CD19^+^CD27^-^) B cells, memory (CD19^+^CD27^+^) B cells and plasma cells (CD19^low^ CD27^high^) expressing CXCR4 are given. Box plots show the median values, 25th and 75th quartile and the range of values. **(C)** The geometric MFI of CXCR4 was evaluated for all aforementioned B-cell subsets. *P < .05, **P < .005 and ***P < .0005 compared with whole blood-gated B cells. Click here for file

Additional file 3: Figure S3 Sample processing modulates membrane CXCR7 expression. **(A)** Surface expression of CXCR7 on CD19^+^-gated B cells from 4 independent healthy Caucasian women was determined by flow cytometry using the unconjugated 9C4 (empty histograms) or isotype control (filled histograms) mAb followed by a PE-conjugated goat anti-mouse F(ab’)_2_ Ab. Displayed data are representative plots of the MFI of CXCR7 at the surface of total B cells obtained either after staining on whole blood (Blood) or fresh (Ficoll) or cryopreserved (Cryo) PBMC. **(B)** The percentage of total (CD19^+^) B cells, naive (CD19^+^ CD27^-^) B cells, memory (CD19^+^ CD27^+^) B cells and plasma cells (CD19^low^ CD27^high^) expressing CXCR7 are given. Box plots show the median values, 25th and 75th quartile and the range of values. **(C)** The geometric MFI of CXCR7 was evaluated for all B-cell subsets. *P < .05 and **P < .005 compared with whole blood-gated B cells. (PPT 945 kb) Click here for file
