# Supplementary figures and images for: Expression of CXCL12 receptors in B cells from Mexican Mestizos patients with systemic lupus erythematosus
Source: J Transl Med. 2012 Dec 18;10:251. doi: 10.1186/1479-5876-10-251 (PMC3571925; doi:10.1186/1479-5876-10-251)

## Slide 1
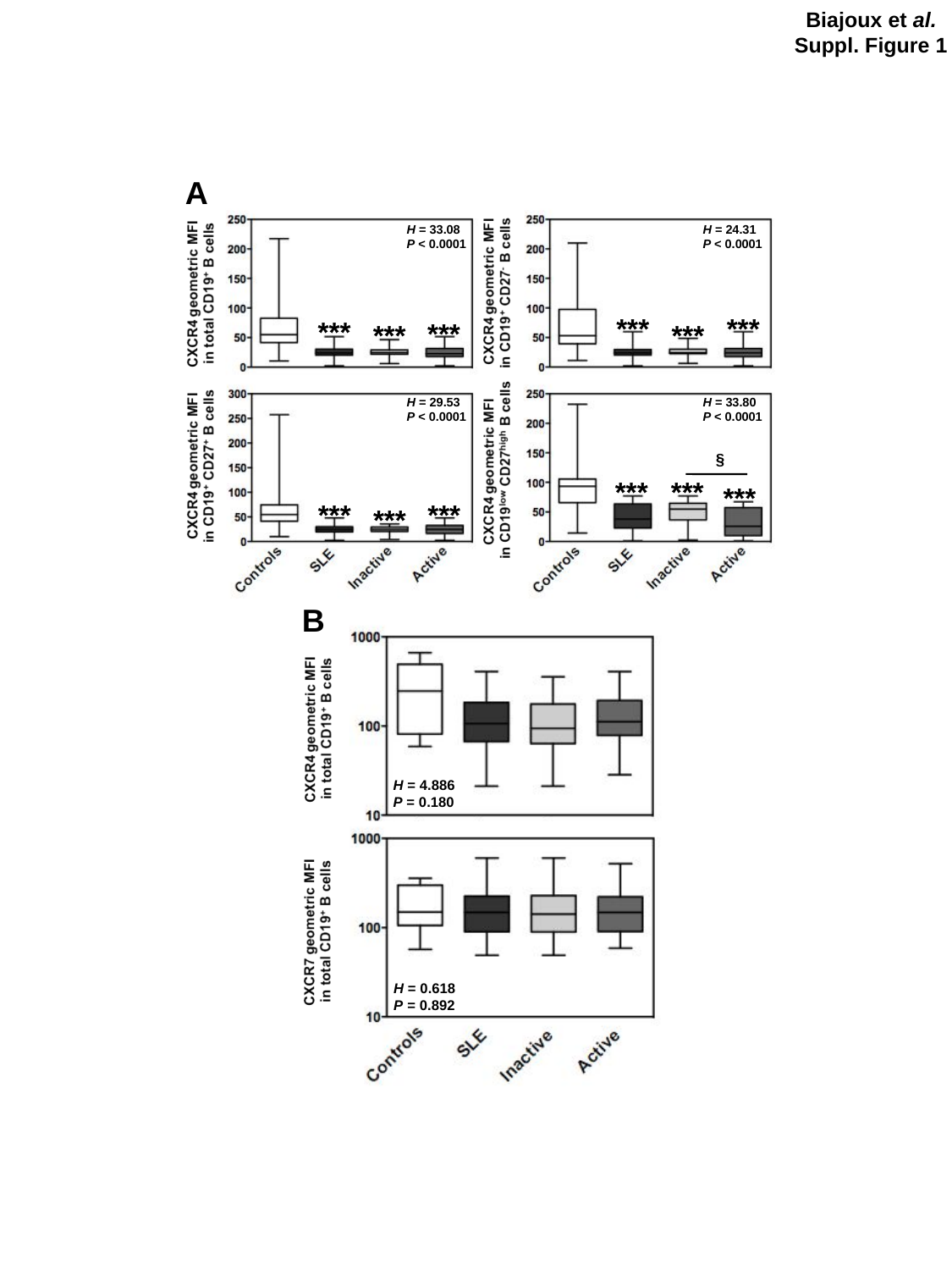

Biajoux et al.
Suppl. Figure 1
A
H = 33.08
P < 0.0001
H = 24.31
P < 0.0001
***
***
***
***
***
***
H = 29.53
P < 0.0001
H = 33.80
P < 0.0001
§
***
***
***
***
***
***
B
H = 4.886
P = 0.180
H = 0.618
P = 0.892

Supplement: Additional file 1: Figure S1 — Distribution of CXCR4 and CXCR7 in SLE B cells. (A) Expression of CXCR4 on CD19+-gated PBMC from SLE patients (n = 41), distributed according to disease activity, i.e. inactive (n = 17) versus active (n = 24), and healthy individuals (n = 45) was determined by flow-cytometric analysis (FACSCalibur, BD Biosciences) using the APC-conjugated 12G5 mAb. The geometric mean fluorescence intensity (MFI) of CXCR4 at the surface of total (CD19+) B cells, naive (CD19+CD27-) B cells, memory (CD19+CD27+) B cells and PC (CD19lowCD27high) from healthy and SLE subjects are displayed. (B) Total pools of CXCR4 and CXCR7 were detected by flow cytometry by staining fixed and permeabilized leukocytes with 9C4 (PE) and 12G5 (APC) mAbs. The geometric MFI of CXCR4 or CXCR7 was evaluated in total B cells. Box plots show the median values, 25th and 75th quartile and the range of values. Kruskal-Wallis H test and associated P values are indicated. ***P < .0005 compared with control B cells. §P < .05 compared with B cells from patients with inactive SLE (as determined using the Mann–Whitney U-test). [file 1479-5876-10-251-S1.ppt]

## Slide 1
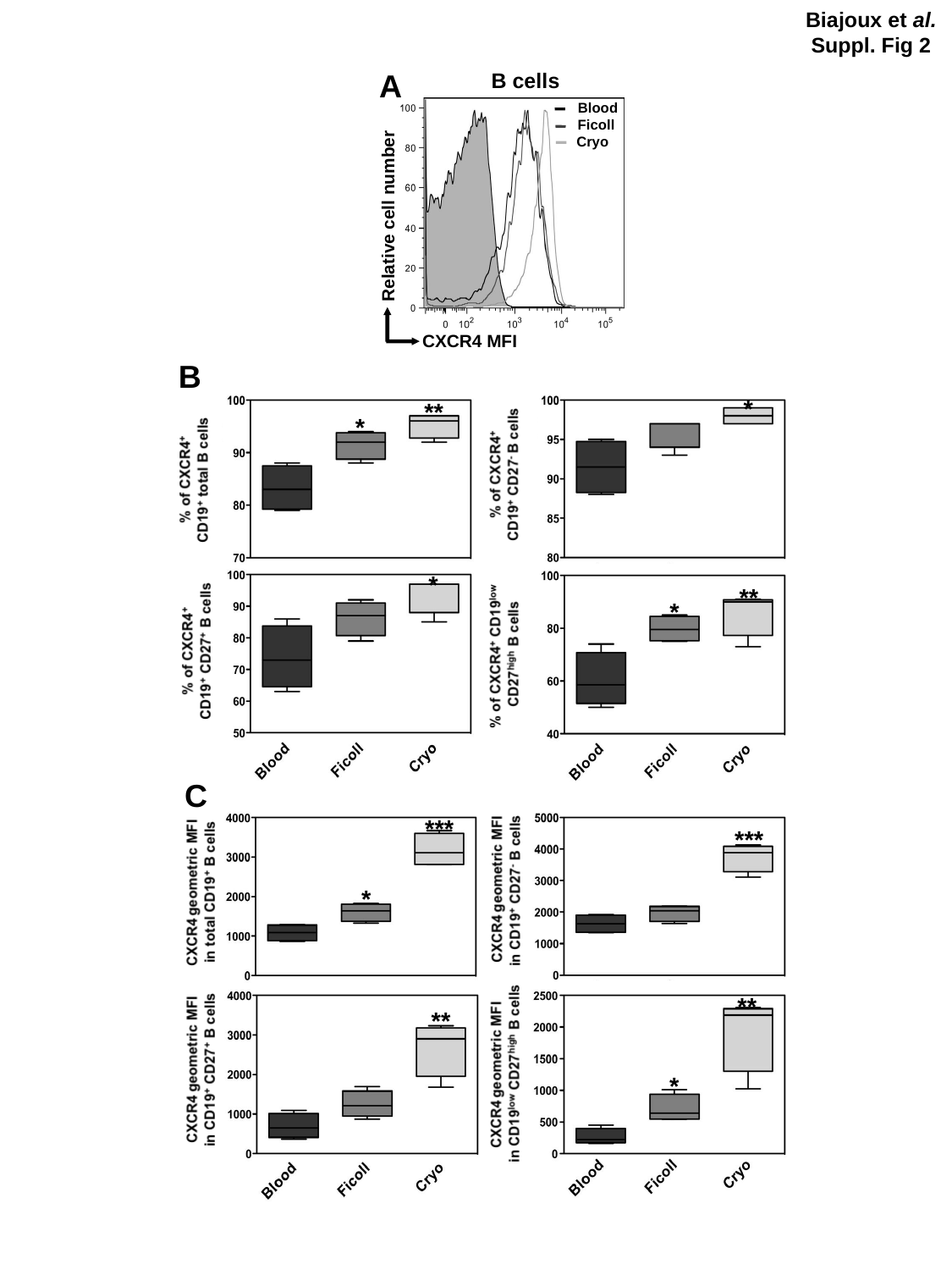

Biajoux et al.
Suppl. Fig 2
A
Blood
Ficoll
Cryo
Relative cell number
CXCR4 MFI
B cells
B
*
**
*
*
**
*
C
***
***
*
**
**
*

Supplement: Additional file 2: Figure S2 — Sample processing modulates membrane CXCR4 expression. (A) Membrane expression of CXCR4 on control CD19+-gated B cells was determined by flow-cytometric (FACS Fortessa, BD Biosciences) analysis using the PE-conjugated 12G5 (empty histograms) or isotype control (filled histogram) mAb. Displayed data are representative plots of the MFI of CXCR4 at the surface of total B cells from 4 independent healthy Caucasian women (age median 35 years, range: 24 to 47 years) obtained either after staining on whole blood (Blood) or fresh (Ficoll) or cryopreserved (Cryo) PBMC. (B) The percentage of total (CD19+) B cells, naive (CD19+CD27-) B cells, memory (CD19+CD27+) B cells and plasma cells (CD19low CD27high) expressing CXCR4 are given. Box plots show the median values, 25th and 75th quartile and the range of values. (C) The geometric MFI of CXCR4 was evaluated for all aforementioned B-cell subsets. *P < .05, **P < .005 and ***P < .0005 compared with whole blood-gated B cells. [file 1479-5876-10-251-S2.ppt]

## Slide 1
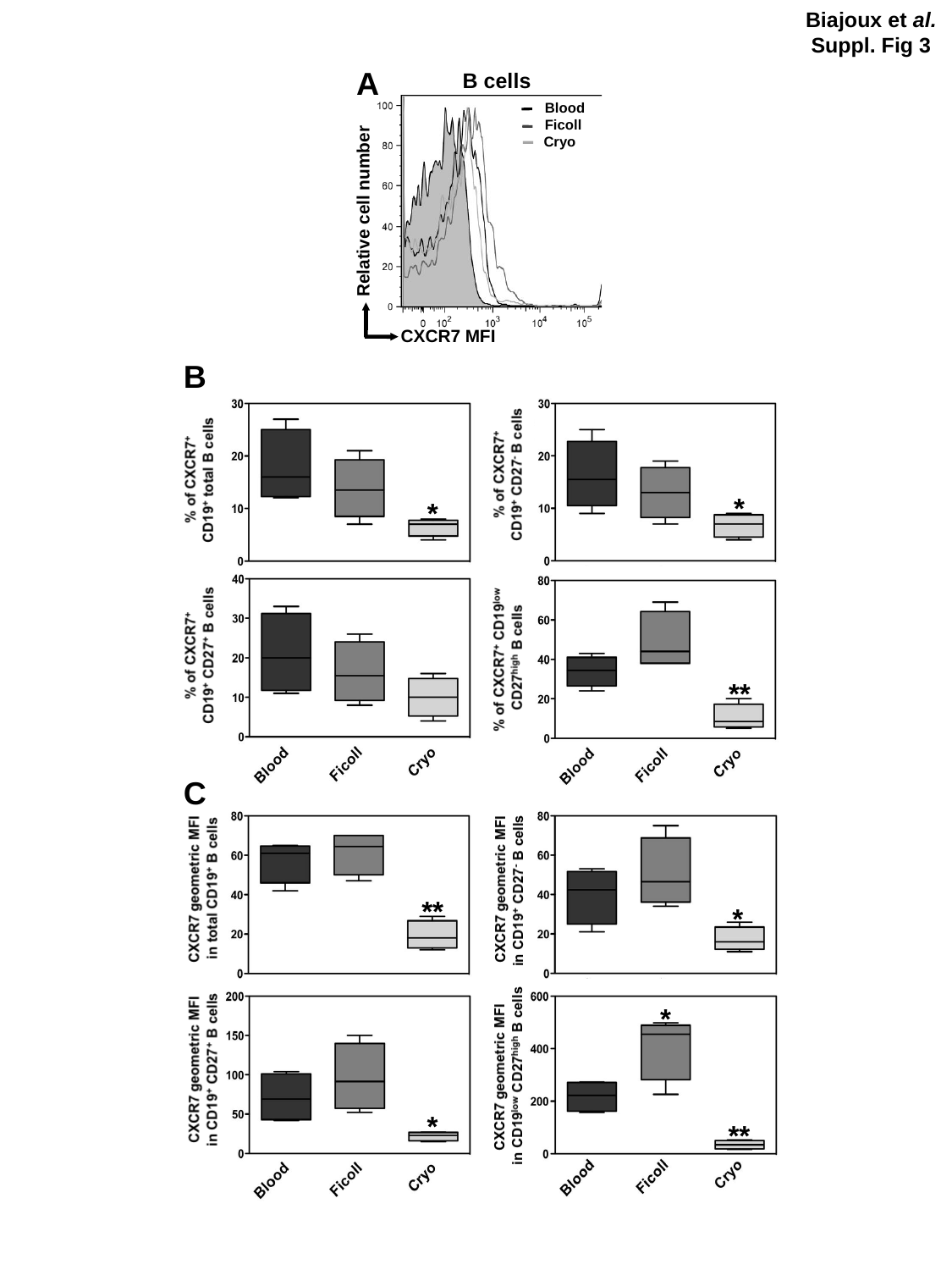

Biajoux et al.
Suppl. Fig 3
A
Blood
Ficoll
Cryo
Relative cell number
CXCR7 MFI
B cells
B
*
*
**
C
**
*
*
*
**

Supplement: Additional file 3: Figure S3 — Sample processing modulates membrane CXCR7 expression. (A) Surface expression of CXCR7 on CD19+-gated B cells from 4 independent healthy Caucasian women was determined by flow cytometry using the unconjugated 9C4 (empty histograms) or isotype control (filled histograms) mAb followed by a PE-conjugated goat anti-mouse F(ab’)2 Ab. Displayed data are representative plots of the MFI of CXCR7 at the surface of total B cells obtained either after staining on whole blood (Blood) or fresh (Ficoll) or cryopreserved (Cryo) PBMC. (B) The percentage of total (CD19+) B cells, naive (CD19+ CD27-) B cells, memory (CD19+ CD27+) B cells and plasma cells (CD19low CD27high) expressing CXCR7 are given. Box plots show the median values, 25th and 75th quartile and the range of values. (C) The geometric MFI of CXCR7 was evaluated for all B-cell subsets. *P < .05 and **P < .005 compared with whole blood-gated B cells. (PPT 945 kb) [file 1479-5876-10-251-S3.ppt]
